# Analysis of Age as a Possible Prognostic Factor for Transcanalicular Multidiode Laser Dacryocystorhinostomy

**DOI:** 10.1155/2014/913047

**Published:** 2014-06-09

**Authors:** Emre Ayintap, Ibrahim Bulent Buttanri, Fariz Sadıgov, Didem Serin, Mustafa Ozsutcu, Julide Canan Umurhan Akkan, Kemal Tuncer

**Affiliations:** ^1^Department of Ophthalmology, Faculty of Medicine, Bezmialem Vakif University, Fatih, 34093 Istanbul, Turkey; ^2^Haydarpaşa Numune Education and Research Hospital, Eye Clinic, 34668 Istanbul, Turkey

## Abstract

*Purpose*. To assess the prognostic value of age on the outcome of transcanalicular multidiode laser dacryocystorhinostomy (TCL-DCR) in patients with acquired nasolacrimal duct obstruction (NLDO). *Methods*. The medical records of TCL-DCR performed between March 2009 and September 2013 were reviewed retrospectively. Inclusion criteria include over 20 years of age, similar mean follow-up period, and similar mean duration of stenting. The main outcome is surgical success. The effect of age on success rate is also evaluated. *Results*. The anatomical success was 52% in Group 1 (20–30 years), 56% in Group 2 (31–40 years), 64% in Group 3 (41–50 years), 76% in Group 4 (51–60 years), and 88% in Group 5 (over 60 years). The statistical difference among Group 1 and Group 5, in terms of surgical success rate, was found to be significant (*P* = 0.009). Additionally, the 20–30-year-old patients had a failure rate 6.76 times higher than that of the over-60-year-old patients (*P* = 0.009; 95% CI, 1.605–28.542). *Conclusion*. TCL-DCR is a surgical treatment option for NLDO for which a skin incision can be avoided. The success rate of TCL-DCR for younger population is lower when compared with elderly population.

## 1. Introduction

The standard surgical approach for the treatment of nasolacrimal duct obstruction (NLDO) or distal canalicular obstruction is external dacryocystorhinostomy (EX-DCR) [1, 2]. EX-DCR is also effective in revision surgeries [3]. However, the introduction of newly developed technologies and imaging techniques for endoscopic approaches, such as endonasal dacryocystorhinostomy (EE-DCR), transcanalicular multidiode laser dacryocystorhinostomy (TCL-DCR), and endocanalicular endoscopic procedures like Laser-Dacryoplastic or Microdrill-Dacryoplastic, have resulted in high success rates when used by experienced practitioners [4–7].

Both EE-DCR and TCL-DCR have the advantages of no skin incision and the possibility of no scarring of the skin wound. Both approaches preserve lacrimal pump function by keeping the medial canthal tendon intact, and both have lower bleeding rates and shorter surgical and patient rehabilitation times [8–11]. Compared with EE-DCR, TCL-DCR has a shorter surgical time, lower bleeding incidence due to the coagulative effect of the laser, and better patient management under local anesthesia, as well as being easier for the surgeon to learn [4, 10, 12, 13]. The success rate of EX-DCR is over 90% [1, 2, 14]. There are relatively few studies on TCL-DCR, and the reported success rates vary from 52% to 96% [9, 10, 13, 15–18]. Although the surgical procedures described in these studies are similar, the wide range of success rates is questionable.

Previous studies were conducted to compare the patient groups with narrow range of age and concluded that presence of silicon tube, time of silicon tube removal, and postoperative follow-up period were found to be closely related to the success rate of TCL-DCR [4, 9, 12, 13, 15, 17, 19]. We think that comparing patient groups with similar age could not represent an appropriate study population to show the age effect on the success rate of the surgery. Accordingly, we designed the current study to find the influence of age on the success rate of TCL-DCR by standardizing the acquired NLDO patients in gender, duration of stent left in situ, and the duration of postoperative follow-up.

## 2. Materials and Methods

The medical records of patients over 20 years of age with acquired NLDO who underwent TCL-DCR with silicone tube intubation surgery in two different centers (Bezmialem Vakif University, Department of Ophthalmology and Haydarpaşa Numune Education and Research Hospital, Eye Clinic, Istanbul) between March 2009 and September 2013 were reviewed retrospectively. Patients with follow-up periods less than six months and silicone stent removal before 8 weeks were excluded from the study.

In order to determine whether there is a relationship between patient age and surgical success rate, the same number of patients with same male/female ratio, similar mean follow-up period, and similar mean duration of stenting were selected into groups for all decades: Group 1, 20–30 years of age; Group 2, 31–40 years of age; Group 3, 41–50 years of age; Group 4, 51–60 years of age; and Group 5, over 60 years of age. The study was carried out in agreement with the Declaration of Helsinki and the ethics committee approved the study. All of the patients provided signed informed consent and were thoroughly informed about the advantages and disadvantages of all available surgical interventions for NLDO.

Indication for surgery was epiphora, due to complete acquired NLDO. In each case, the diagnosis of NLDO was based on the result of lacrimal irrigation performed preoperatively. All of the patients had undergone preoperative endoscopic nasal evaluation, and those with nasal pathologies such as polyps, mass lesions, scars, or advanced septal deviations were not enrolled. None of the patients had previously undergone lacrimal surgery, and none had any history of nasoorbital trauma.

## 3. Surgical Technique

All of the surgeries were performed under local anesthesia. The approximate surgical time of each case was 20 minutes. Intranasal anesthesia and vasoconstriction were achieved by tamponading the nasal cavity with cotton sponges soaked in a 1 : 1 mixture of epinephrine 1 : 100,000 and 4% lidocaine. After dilatation of the superior and inferior lacrimal canaliculi with punctum dilators, 600-*μ*m quartz Teflon fiber was inserted through the inferior lacrimal canaliculus with a red pilot beam light activated.

Through a nasal endoscope, the pilot beam transillumination from the lacrimal sac was recognized and adjusted anteroinferiorly at the insertion point of the middle concha. Then, the laser beam was activated to create a fistula between the lacrimal sac and the nasal cavity. The laser used was the Multidiode SLPTM S30 Gallium Arsenide P diode laser (Intermedic, Lower Saxony, Germany) with a repetitive pulse mode of 980 nm. The laser settings were as follows: power 10 W, pulse length 90 ms, and pause between pulses 50 ms. Once the fistula was created, its orifice was enlarged at the endonasal side with additional laser pulses to reach a width of 6–10 mm. After removal of the laser fiber, lacrimal system irrigated with fluorescein-stained saline and then silicone intubation was performed, followed by tobramycin/dexamethasone eye drops four times daily and oral antibiotics twice daily for one week after surgery. The patients were evaluated by the operating surgeons on the first day, first week, second week, and first, third, sixth, 12th, 18th, and 24th months (routine follow-up protocol) following surgery. We defined surgical success as resolution of symptoms (epiphora or discharge) and unobstructed lacrimal irrigation.

## 4. Statistical Analysis

Statistical analyses between groups were performed for patient gender, mean follow-up period, mean duration of stent left in situ, and surgical success rate. Statistical analyses were conducted with the Statistical Package for Social Sciences for Windows 17.0 program (SPSS, Chicago, IL). During the evaluation of the data, Pearson's Chi-square, One way Anova and Independent Samples *t*-test were used. Logistic regression analysis was used to reveal any independent risk factors. A *P* value of less than 0.05 was considered significant with a 95% confidence interval (CI).

## 5. Results

A total of 125 patients were enrolled in the study. Both of the groups consisted of 25 patients. The mean age of the Group 1, Group 2, Group 3, Group 4, and Group 5 was 25.4 ± 2.6; 35.0 ± 2.4; 45.3 ± 2.2; 55.6 ± 2.2; and 67.4 ± 3.1 years, respectively. The mean durations of stent left in situ were Group 1, 17.1 ± 2.7, Group 2, 16.9 ± 3.1, Group 3, 17.5 ± 2.5, Group 4, 17.6 ± 2.7, and Group 5, 17.0 ± 2.1 weeks. The mean follow-up periods were Group 1, 15.5 ± 8.3 months; Group 2, 16.8 ± 5.6 months; Group 3, 16.2 ± 7.1 months; Group 4, 16.6 ± 7.6 months; and Group 5, 15.3 ± 6.9 months. There were no statistically significant differences among groups in terms of patient numbers, genders, mean duration of stent left in situ (*P* = 0.924), or mean follow-up period (*P* = 0.852).

Surgical success was achieved in 13 of the 25 patients (52%) in Group 1, 14 of the 25 patients (56%) in Group 2, 16 of the 25 patients (64%) in Group 3, 19 of the 25 patients (76%), and 22 of the 25 patients (88%) in Group 5, at the end of the follow-up periods (Table 1). The statistical difference between Group 1 and Group 5 in terms of surgical success rate was found to be significant (*P* = 0.009). Further, these data were entered into the multivariate logistic regression analyses and it was found that the 20–30-year-old patients had a failure rate 6.76 times higher than that of the over 60-year-old patients (*P* = 0.009; 95% CI, 1.605–28.542).

Surgery was found to be successful in 84 (67.2%) of all patients. When we compared them with the patients without successful results, there were no significant differences between the groups by means of gender, mean follow-up time, and the mean duration of stent left in situ. However, there was significant difference between the mean age of the successful patients (49.0 ± 14.3 years) and the mean age of the failed patients (38.53 ± 13.0 years) (*P* = 0.01) (Table 2).

## 6. Discussion

A wide range of success rates with TCL-DCR have been reported. One of the main differences between these studies was the mean age of the study groups. Gupta et al. had a 90.5% success rate of TCL-DCR in patients with a mean age of 30.1 years (range: 15–69) [19]. Derya et al. reported an 86% success rate in 29 patients with a mean age of 45.24 years [20]. In the study of 122 patients with a mean age of 59 years (range: 13–84), Drnovšek-Olup and Beltram had a success rate of 83.3% [9]. Plaza et al. performed ETL-DCR on 25 patients with a mean age of 57.3 years and had an 88% success rate [21]. Our overall success rate with TCL-DCR was 67.2% in 125 patients with a mean age of 45.6 years. In our study, the patients were divided into age groups for all decades, with the aim of achieving more precise statistical results and it was found that the 20–30-year-old patients had a failure rate 6.76 times higher than that of the over 60-year-old patients. In our study the overall success rate of 67.2% is quite low. Probably higher proportion of younger patients is the reason for this result.

The main disadvantage of TCL-DCR is that it creates a smaller bony window than the one which EX-DCR does; therefore, the main cause of failure with this technique is the obstruction of the bony window due to fibrovascular proliferation. Henson RD et al. reported a success rate of 92.8% with multiple postoperative applications of Mitomycin C [22]. Other reasons for failure of TCL-DCR are adhesions between the rhinostomy and the middle concha [12, 16, 23–25]. Old patients mostly have involutional NLDO; however, young patients tend to have local or systemic causes to precipitate NLDO. It has also been reported that strong expression of nasal mucosal heat shock protein 47 leads to the induction of fibrosis and scar tissue, thus decreasing the effectiveness of EE-DCR [26]. As age increases, the number of fibroblasts decreases, due to the degeneration process. Decreased numbers and activity of fibroblasts produce fewer fibrous components. Posterior capsule opacification, commonly seen after cataract surgery in young patients, has a similar mechanism. Human lens epithelial cells (HLEC) proliferate by the induction of basic fibroblast growth factor. HLEC proliferation decreases as age increases; thus, the incidence rate of posterior capsule opacification is high in children [27]. All of these processes may explain the high failure rate of TCL-DCR in young population.

Linberg et al. reported that the osteotomy size created by an EX-DCR in 22 cases was 11.84 mm in diameter at the time of surgery and decreased to an average size of 1.80 mm [28]. In an ultrasonic assessment of osteotomy size following EX-DCR, Ezra et al. reported a significant reduction in osteotomy size after two weeks and after six months [29]. TCL-DCR creates a smaller bony window than the one which EX-DCR does. In vitro and in vivo studies have revealed that bone fractures heal more quickly in younger individuals than in adults because of their superior osteoblastic activity; therefore, the possibility of satisfactory results after TCL-DCR is lower in patients under 40 years of age, who have higher fibroblastic and osteoblastic activity. The results of our study support this hypothesis.

The main reason for the ongoing popularity of TCL-DCR is the absence of any incision marks. Sharma et al. performed 263 EX-DCR operations and reported significant scar tissue at the incision area in 19.4% of the patients and these scars caused cosmetic discomfort in 10.3% [8]. According to our results, age is a prognostic factor for TCL-DCR outcomes and success rate of TCL-DCR operation is lower in young patients. Therefore, it is important to inform patients thoroughly about the success rate of this technique in the younger population.

## Figures and Tables

**Table 1:** Comparison of the characteristics and the success rate of the TCL-DCR in different age groups.

	Group 1 (20–30)	Group 2 (31–40)	Group 3 (41–50)	Group 4 (51–60)	Group 5 (over 60)	*P* value
Number of cases	25	25	25	25	25	
Male : female ratio	8 : 17	8 : 17	8 : 17	8 : 17	8 : 17	
Mean age ± SD (years)	25.4 ± 2.6	35.0 ± 2.4	45.3 ± 2.2	55.6 ± 2.1	67.4 ± 3.1	
Number of successful cases	13 (52%)	14 (56%)	16 (64%)	19 (76%)	22 (88%)	0.041^a^
Mean duration of stent left in situ ± SD (weeks)	17.1 ± 2.7	16.9 ± 3.1	17.5 ± 2.5	17.6 ± 2.7	17.0 ± 2.1	0.924^b^
Mean follow-up time (months)	15.5 ± 8.3	16.8 ± 5.6	16.2 ± 7.1	16.6 ± 7.6	15.3 ± 6.9	0.852^b^

^a^Pearson Chi-square test.

^b^One way Anova test.

**Table 2:** Comparison of the success rate of the TCL-DCR in different age groups.

	Successful patients	Failed patients	*P* value
Number of cases (percentage)	84 (67.2%)	41 (32.8%)	
Mean age ± SD (years)	49.0 ± 14.3	38.53 ± 13.0	0.01^c^
Male : female ratio	26 : 58	14 : 27	0.826^c^
Mean duration of stent left in situ ± SD (weeks)	16.9 ± 2.4	17.7 ± 3.0	0.223^c^
Mean follow-up time (months)	16.1 ± 6.8	16.9 ± 7.6	0.872^c^

^c^Independent Samples *t*− test.
